# Wild-Type *Hras* Suppresses the Earliest Stages of Tumorigenesis in a Genetically Engineered Mouse Model of Pancreatic Cancer

**DOI:** 10.1371/journal.pone.0140253

**Published:** 2015-10-09

**Authors:** Jamie D. Weyandt, Benjamin L. Lampson, Sherry Tang, Matthew Mastrodomenico, Diana M. Cardona, Christopher M. Counter

**Affiliations:** 1 Department of Pharmacology & Cancer Biology, Duke University Medical Center, Durham, North Carolina, United States of America; 2 Department of Pathology, Duke University Medical Center, Durham, North Carolina, United States of America; 3 Department of Radiation Oncology, Duke University Medical Center, Durham, NC, United States of America; Klinikum rechts der Isar der TU München, GERMANY

## Abstract

Oncogenic, activating mutations in *KRAS* initiate pancreatic cancer. There are, however, two other *Ras* family members, *Nras* and *Hras*, which can be activated in the presence of oncogenic *Kras*. The role of these wild-type *Ras* proteins in cancer remains unclear, as their disruption has been shown to enhance or inhibit tumorigenesis depending upon the context. As pancreatic cancer is critically dependent upon *Ras* signaling, we tested and now report that loss of *Hras* increases tumor load and reduces survival in an oncogenic *Kras*-driven pancreatic adenocarcinoma mouse model. These effects were traced to the earliest stages of pancreatic cancer, suggesting that wild-type *Hras* may suppress tumor initiation. In normal cells, activated *Ras* can suppress proliferation through p53-dependent mechanisms. We find that the tumor suppressive effects of *Hras* are nullified in a homozygous mutant p53 background. As such, loss of wild-type *Hras* fosters the earliest stages of pancreatic cancer in a p53-dependent manner.

## Introduction

It is estimated that approximately 1.5% of Americans will develop pancreatic cancer, primarily *p*ancreatic *d*uctal *a*deno*c*arcinoma (PDAC). The vast majority of these individuals will succumb to this disease, with a five-year survival rate of only 6.7% [1]. Thus, it is critical to elucidate the signaling pathways underlying this incredibly deadly cancer. Activating mutations in *KRAS* are the most common genetic alteration found in human PDAC, being present in over 90% of all cases. In fact, the Ras pathway is argued to be engaged in essentially all PDAC [2].

*KRAS*, and the other Ras family members *NRAS* and *HRAS*, encode highly related small GTPases. Normally, stimulation of guanine nucleotide exchange factors [GEFs] promotes the conversion of Ras from an inactive, GDP-bound state to an active, GTP-bound state. Ras-GTP binds to and activates effector proteins that engage the MAPK, PI3K, RalGEF, and other signaling pathways involved in cellular growth and survival. GTPase-activating proteins (GAPs) then enhance hydrolysis of GTP and revert Ras back to an inactive state [3].

The somatic mutations detected in the *KRAS* gene inactivate the endogenous or GAP-stimulated GTPase activity of the encoded protein, resulting in constitutively GTP-bound and activated Kras [3]. In addition to being commonly detected in PDAC [2], these mutations are also found in the earliest stages of this disease [4] and, when introduced into the murine *Kras* gene, induce early stage pancreatic cancer that can progress to frank PDAC [5]. As such, oncogenic mutations in *KRAS* are thought to be the driver mutations in PDAC.

Although the involvement of the oncogenic mutant form of Kras in pancreatic cancer is well described, the remaining wild-type Ras isoforms may also be activated. The first hints in this regard were the findings that targeting the negative regulator RasGAP to the plasma membrane [6] or reducing wild-type Nras expression [7] dampened oncogenic Hras signaling. Indeed, wild-type Nras and Hras have been found to be activated downstream of oncogenic Kras in a manner dependent upon *S*-nitrosylation [8] or expression of the GEF son-of-sevenless [SOS] [9]. Moreover, wild-type Ras proteins are still susceptible to activation by growth factors, even in the presence of oncogenic Kras [10].

Wild-type Ras proteins can have divergent effects depending on the setting. On one hand, they can inhibit tumorigenesis. Specifically, oncogenic Ras can induce a senescent growth arrest when expressed in normal cells [11] and inhibit tumor formation [12], suggesting a tumor suppressive role for the wild-type Ras proteins. In agreement, carcinogen-induced oncogenic mutations in *Kras* or *Hras* in lung and skin tumors of mice are often accompanied by loss of the reciprocal wild-type allele [13–15]. Similarly, loss of heterozygosity of the oncogenic *RAS* gene has been reported in human cancers [16]. Moreover, mice lacking one allele of wild-type *Kras* [15], or both alleles of either *Hras* or *Nras*, develop more *Kras* mutation-positive lung tumors [17]. A tumor-suppressive role for wild-type *Nras* was also observed in a mouse model of thymic lymphomas driven by oncogenic *Nras* [18]. Thus, wild-type Ras proteins can be tumor suppressive, especially in settings of early tumorigenesis.

Wild-type Ras proteins can also foster tumorigenesis. Specifically, mice lacking one or both alleles of wild-type *Hras* or *Nras* develop fewer carcinogen-induced *Hras* mutation-positive skin tumors [17]. Knocking down the expression of wild-type Ras proteins [8, 19], or reducing their activation by eNOS [8] or SOS [9], inhibits cell viability, transformation, and/or tumorigenic growth of *KRAS* mutation-positive human cancer cell lines. Conversely, stimulation of wild-type Ras proteins by EGF promotes proliferation of such cells [10]. Thus, particularly in models of more advanced disease, wild-type Ras proteins can enhance tumorigenesis.

Given that wild-type Ras proteins can be activated downstream of oncogenic Kras, yet give rise to opposite effects on tumorigenesis depending on the context, it is important to ascertain their role in the cancer most frequently associated with *KRAS* mutations, namely pancreatic. Thus, we assessed the consequence of disrupting the endogenous, wild-type *Hras* gene on the development of oncogenic *Kras*-driven tumorigenesis in the pancreas of mice. We report that loss of wild-type *Hras* promotes tumorigenesis in this model, suggesting a tumor suppressive role for wild-type *Hras* at the early stages of pancreatic cancer.

## Materials and Methods

### Mouse pancreatic cancer models

*Kras* ^*LSL-G12D/+*^, *Trp53* ^*LSL-R172/+*^, and *Pdx-1-Cre* ^*tg/+*^ mice were obtained from Jackson Labs and as a kind gift from David Kirsch. *Hras* ^*-/-*^ mice were a kind gift from the NCI and Eugenio Santos. *Kras* ^*LSL-G12D/+*^ *;Hras* ^*+/-*^ and *Pdx-1-Cre* ^*tg/+*^ *;Hras* ^*+/-*^ mice were bred to generate *Hras* ^*+/+*^ and *Hras* ^*-/-*^ KC (*LSL-Kras* ^*G12D/+*^ *;Pdx-1-Cre* ^*tg/+*^) littermates. *Kras* ^*LSL-G12D/+*^ *;Trp53* ^*LSL-R172/+*^ *;Hras* ^*+/+*^ and *Pdx-1-Cre* ^*tg/+*^ *;Hras* ^*+/+*^ mice were bred to generate *Hras* ^*+/+*^ KPC (*LSL-Kras* ^*G12D/+*^ *;Trp53* ^*LSL-R172/+*^ *;Pdx-1-Cre* ^*tg/+*^) mice. *Kras* ^*LSL-G12D/+*^ *;Trp53* ^*LSL-R172/+*^ *;Hras* ^*-/-*^ and *Pdx-1-Cre* ^*tg/+*^ *;Hras* ^*-/-*^ mice were bred to generate *Hras* ^*-/-*^ KPC mice. *Kras* ^*LSL-G12D/+*^ *;Trp53* ^*LSL-R172/+*^ *;Hras* ^*+/+*^ and *Pdx-1-Cre* ^*tg/+*^ *;Trp53* ^*LSL-R172/+*^ *;Hras* ^*+/+*^ mice were bred to generate *Hras* ^*+/+*^ KPPC (*LSL-Kras* ^*G12D/+*^ *;Trp53* ^*LSL-R172/ LSL-R172H*^ *;Pdx-1-Cre* ^*tg/+*^) mice. *Kras* ^*LSL-G12D/+*^ *;Trp53* ^*LSL-R172/+*^ *;Hras* ^*-/-*^ and *Pdx-1-Cre* ^*tg/+*^ *;Trp53* ^*LSL-R172/+*^ *;Hras* ^*-/-*^ mice were bred to generate *Hras* ^*-/-*^ KPPC mice. The described mice were monitored for health and weight three times per week and were euthanized at either the indicated fixed time points or upon reaching a moribundity endpoint. Moribundity endpoints were defined as weight loss exceeding 15% of total body weight, indications of abdominal ascites or swelling, or signs of pain or distress. This study was carried out in strict accordance with the recommendations in the Guide for the Care and Use of Laboratory Animals of the National Institutes of Health. A protocol for this study was specifically approved by the Institutional Animal Care and Use Committee at Duke University (protocol A279-13-11).

### Mouse PDAC cell lines

Pancreatic tumor tissue from three *Hras* ^*+/+*^ and three *Hras* ^*-/-*^ KPC mice was minced in collagenase V (Sigma-Aldrich) for 30 minutes at 37°C, and resultant cells were cultured in Dulbecco's Modified Eagle's Media (DMEM) + 10% FBS for at least 4 passages.

### Quantification of normal pancreatic acinar area

The percentage of normal acinar area was quantified at 8 and 36 week time-points in KC mice in a blinded study of 4–5 randomly selected high-power fields of H & E-stained pancreatic section per mouse. The amount of acinar area per section was expressed as a percentage of the total area of tissue in the field, determined using Adobe Photoshop freehand selection tool.

### Grading of pancreatic lesions

H&E stained sections were blindly reviewed by pathologists (S. Tang, M. Mastrodomenico, and D. Cardona) at 4, 8, and 36 week time points. Each lobule of the pancreas from each section was examined, and the highest grade lesion (ADM or PanIN) from each lobule was identified, and the percent of lobules with the highest grade of each type of lesion was determined based upon the total number of lobules counted.

### ADM immunoflourescent staining

Pancreatic specimens from *KC* mice were snap frozen in liquid nitrogen and 8 uM-thick sections were cut using a cryotome. The samples were fixed with 4% paraformaldehyde and permeabilized in 0.2% Triton-X-100. Samples were blocked in normal donkey serum then incubated with 1:100 dilution of the primary antibodies Goat Anti-Amylase C-20 and sc-12821 Rabbit Anti-CK-19 H-60 (Santa Cruz Biotechnology Inc.) overnight at 4^°^C. Samples were then incubated with the secondary antibodies Alexa fluor 488 Donkey anti-rabbit IgG (Invitrogen A-21206) or Alexa-fluor 594 Donkey anti-goat IgG (Invitrogen A-11058) at a 1:500 dilution for 1 hour at room temperature, then mounted with ProLong Gold antifade reagent with DAPI (Life Technologies P36931). Total number of lesions with cells co-staining for amylase and CK-19 were quantified per high power field in merged images from a Zeiss Axio Imager widefield fluorescence microscope in a blinded fashion.

### Senescence-associated β-galactosidase staining

Snap-frozen pancreatic samples were stained as described by Debacq-Chainiaux *et al* [20] and 5 randomly selected, high-power, images from an Olympus Vanox X microscope were quantified using Image J software in a blinded fashion. Color thresholding was used to determine the total amount of staining per high power field, and the freehand selection tool was used to select only the lesions, where color thresholding was applied to calculate the percentage of positive staining area from only the lesions.

### Ki67, CC3, and p16 immunohistochemistry

Heat-induced antigen retrieval was performed on formalin-fixed, paraffin-embedded sections, followed by staining with an anti-Ki67 primary antibody at a 1:50 dilution (Dako M7249), an anti-CC3 primary antibody at a 1:100 dilution (Cell Signaling, D175), or anti-p16 primary antibody (BD Biosciences, 551153) at a 1:100 dilution. Peroxidase-based detection was performed using Vectastain Elite ABC Kit (Vector labs). The total number of Ki67-positive staining cells per field in each of 5 randomly selected, high-power fields was counted in a blinded fashion, as well as the number of lesions with at least one positive-staining cell and quantified as a percentage of the total number of lesions. For CC3 and p16, 5 randomly selected, high-power fields were quantified in a blinded fashion using Image J software. Color thresholding was used to determine the total amount of CC3 and p16 staining per high-power field, and the freehand selection tool was used to select only the lesions, where color thresholding was applied to calculate the percentage of positive staining area from only the lesions.

### *Hras*-GTP analysis

Early passage (within 5 passages from adaption to culture) *Hras* ^*-/-*^ KPC cell lines were stably infected with retroviruses derived from pBabePuro (vector control) or pBabePuroFLAG-Hras encoding wild-type mouse *Hras* cDNA, and selected with puromycin, as previously described [21]. Stable lines were then tested within 2 passages for Hras GTP levels by affinity capture with the RBD of Raf followed by immunoblot for HRAS with an α-HRAS antibody (Santa Cruz, sc520), as previously described [21].

### PCR of *Kras* alleles

DNA was isolated from pancreatic tissue, facial papillomas, or vulvar tumors and amplified by PCR to detect the wild-type and recombined *Kras* alleles as previously described [5].

### Statistics

Statistical analyses were performed using GraphPad Prism v5 (GraphPad Software). A 2-sided, unpaired *t*-test was used to compare the amount of normal acinar tissue remaining in the pancreata in *Hras* ^*+/+*^ and *Hras* ^*-/-*^ *KC* and *KPPC* mice, and to compare levels of Ki67, p16, ADM, SA-β-gal, and CC3 staining in each cohort. A 2-sided unpaired *t-*test was also used to compare the levels of each type of graded lesions at the specified time-points and the number of facial papillomas between cohorts. These results were then confirmed by Mann-Whitney test using the same software. Kaplan-Meier survival curves were generated for each of the *KPC* and *KPPC* cohorts, as well as for the development of facial and vulvar papillomas over time in the *KPC* cohort, and *P*-values were calculated using the log-rank (Mantel-Cox) test.

## Results

### Increased number of PanIN lesions in the absence of wild-type *Hras*

**Fig 1:**
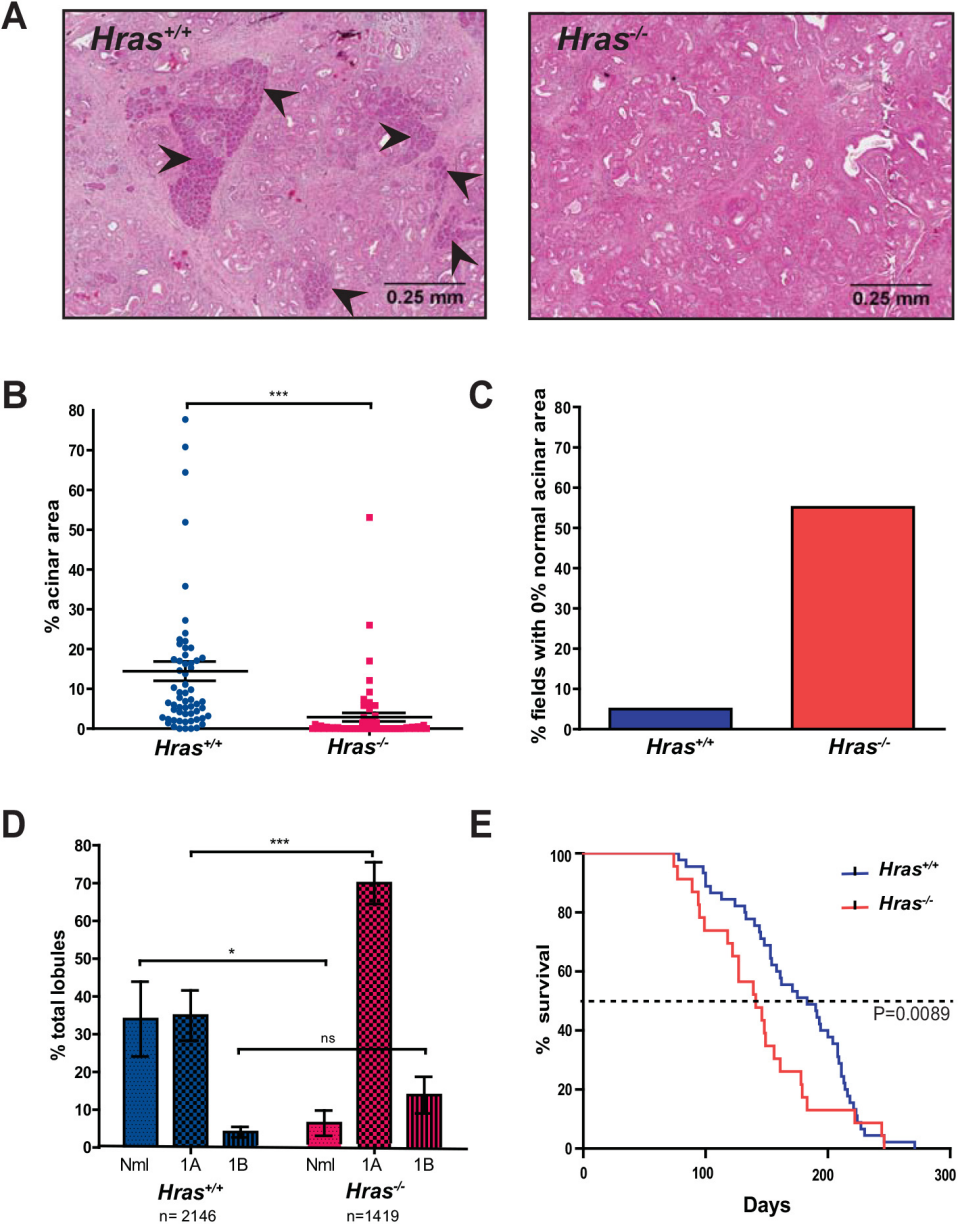
Mice lacking wild-type *Hras* develop more PanIN lesions and have reduced survival in oncogenic *Kras*-driven models of pancreatic cancer. (**A**) Representative H & E stained sections (arrowheads: normal acinar cells), (**B**) quantification of % total normal acinar area remaining per field (at 4x magnification, 4–5 fields from 12 mice, bar: mean ± S.E.M.), (**C**) % of fields (at 4x magnification) with no normal acinar tissue (based on the data from B), and (**D**) % of lobules with the indicated highest grade lesion (Nml: normal, 1A: PanIN-1A, 1B: PanIN-1B, bar: mean ± S.E.M.) of pancreata isolated from *Hras* ^*+/+*^ versus *Hras* ^*-/-*^ KC mice at 9 months of age. (**E**) Kaplan-Meier curve of *Hras* ^*+/+*^ (n = 45) versus *Hras* ^*-/-*^ (n = 23) KPC mice. ns: not significant. *P<0.05. ***P<0.0001.

To assess the role of wild-type Ras proteins on oncogenic *Kras*-driven pancreatic tumorigenesis *in vivo*, mice were generated with either homozygous null *Hras* ^*-/-*^ [22] or wild-type *Hras* ^*+/+*^ alleles in a ***K*** *ras* ^*LSL-G12D/+*^ *;Pdx-1-* ***C*** *re* ^*tg/+*^ (KC) background [5]. We chose Hras, as it is activated downstream of oncogenic Kras in PDAC cell lines [8–10], and of the three *Ras* genes, is the only one that is not embryonic lethal in a background like that of KC mice which encodes only one functional *Kras* allele [22]. We chose the KC model because the activation of an endogenous oncogenic (G12D) *Kras* in the developing pancreas of these mice results in the development of pre-invasive pancreatic intraepithelial neoplasias (PanINs) that progress at a low frequency to PDAC through a series of histological changes that resemble those seen in human patients [5]. Cohorts of 12 *Hras* ^*+/+*^ and *Hras* ^*-/-*^ KC mice were generated and then euthanized at 36 weeks of age, a time when a spectrum of PanIN lesions are easily detected [5]. H & E staining of pancreatic sections revealed a frank loss of normal tissue and expansion of PanIN lesions in the *Hras* ^*-/-*^ KC mice (Fig 1A). Quantification of the amount of normal acinar tissue revealed that *Hras* ^*+/+*^ KC mice had an average of 14.5% normal tissue/field, whereas *Hras* ^*-/-*^ KC mice had 2.9%, a significant decrease of nearly 5-fold in *Hras* ^*-/-*^ KC mice (Fig 1B). This effect was more evident when the percentage of sections lacking normal acinar tissue was quantified. 5% of sections in *Hras* ^*+/+*^ KC mice lacked normal acinar tissue whereas this value was 55.1% in *Hras* ^*-/-*^ KC mice, an increase of over 11-fold in *Hras* ^*-/-*^ KC mice (Fig 1C). Pathological grading of 2146 pancreatic lobes from *Hras* ^*+/+*^ KC mice and 1419 lobes from*Hras* ^*-/-*^ KC mice revealed 27.4% fewer lobes with no lesions and 30% more lobes with the highest grade being PanIN 1A in *Hras* ^*-/-*^ KC mice (Fig 1D). Thus, wild-type *Hras* suppresses the tumorigenic activity of oncogenic *Kras* during pancreatic tumorigenesis.

### Decreased survival in the absence of wild-type *Hras*

Admittedly, the above approach did not determine the effect of *Hras* loss on the commonly diagnosed stage of PDAC [23] or on survival, the most clinically relevant endpoint. To overcome these shortcomings, we generated *Hras* ^*+/+*^ and *Hras* ^*-/-*^ mice in a ***K*** *ras* ^*LSL-G12D/+*^ *;Tr* ***p*** *53* ^*LSL-R172H/+*^ *;Pdx-1-* ***C*** *re* ^*tg/+*^ [KPC] setting [24]. These mice are based on the KC genetic background, but contain an additional inducible dominant-negative *Trp53* ^*R172H*^ allele, which promotes PDAC that pathologically resembles the human disease [24]. Cohorts of 45 *Hras* ^*+/+*^ and 23 *Hras* ^*-/-*^ KPC mice were generated and monitored regularly. Mice were euthanized upon reaching humane moribundity endpoints to determine their lifespan. This analysis revealed that the median survival in *Hras* ^*+/+*^ KPC mice was 183 days, but 141 days in *Hras* ^*-/-*^ KPC mice, a significant reduction of nearly a quarter in *Hras* ^*-/-*^ KPC mice (Fig 1E). Thus, loss of wild-type *Hras* not only potentiates early oncogenic *Kras-*driven pancreatic tumorigenesis, but is also associated with a reduction in lifespan in mice developing PDAC.

### Increased number of skin papillomas in the absence of wild-type *Hras*

**Fig 2:**
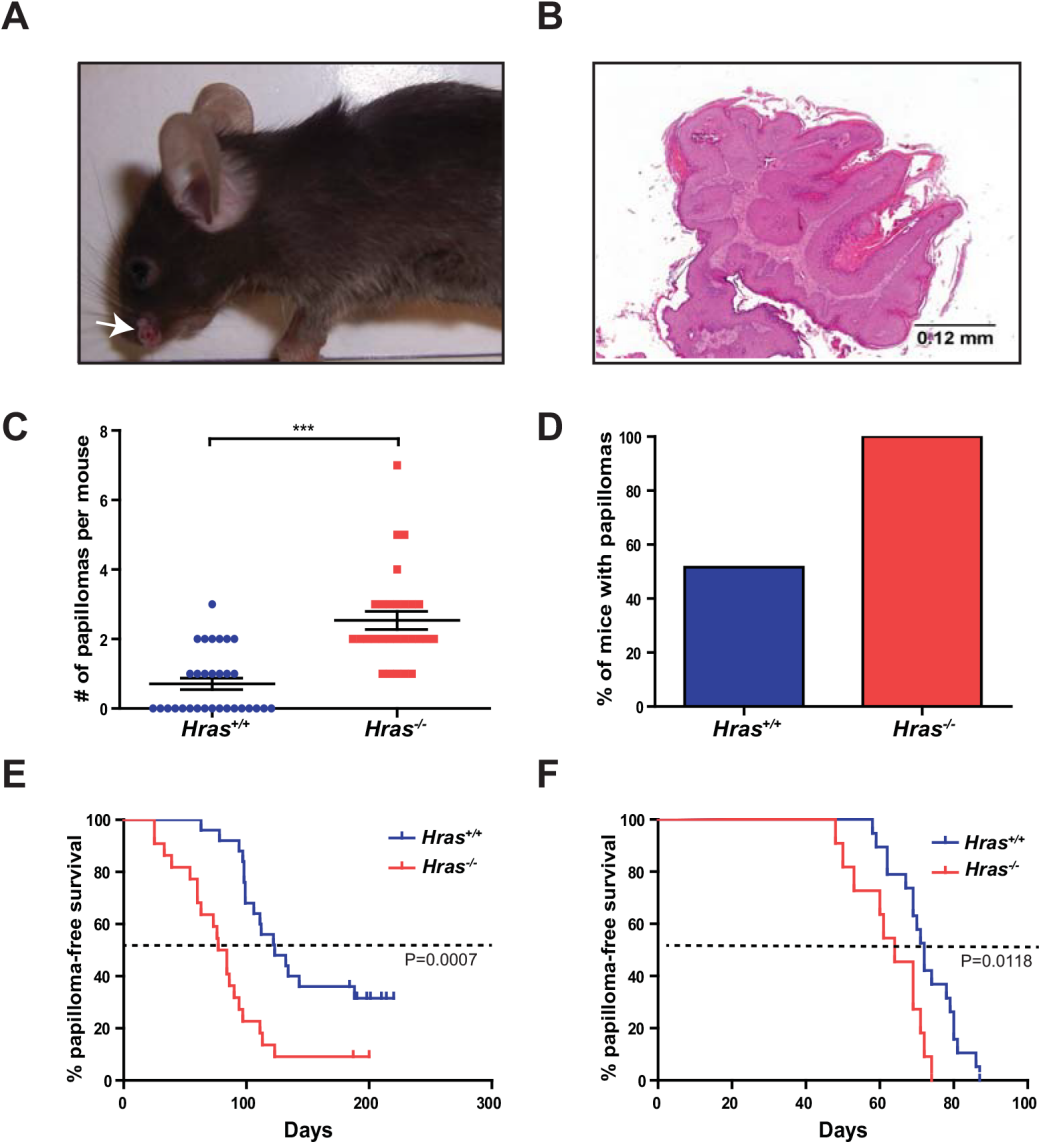
Mice lacking wild-type *Hras* develop more oncogenic Kras*-*driven skin tumors. (**A**) Representative photograph (white arrow) and (**B**) H & E stained section of facial papillomas that develop in KC mice. (**C**) Number of facial papillomas per *Hras* ^*+/+*^ (n = 31) versus *Hras* ^*-/-*^ (n = 28) KC mice (bar: mean ± S.E.M.). (**D**) % of total *Hras* ^*+/+*^ and *Hras* ^*-/-*^ KC mice (from **C**) that developed facial papillomas. (**E**) % facial papilloma-free survival of *Hras* ^*+/+*^ (n = 45) versus *Hras* ^*-/-*^ (n = 23) KPC mice. (**F**) % vulvar papilloma-free survival of *Hras* ^*+/+*^ (n = 19) versus *Hras* ^*-/-*^ (n = 11) female KPC mice. ***P<0.0001.

In addition to PanIN lesions, KC mice are prone to develop skin papillomas in the face and vulvar tissues due to Cre expression by *Pdx-1* in these tissues [5, 25–27]. The development of papillomas in the same KC mice provides a well-controlled system to assess the role of wild-type Hras in a second independent tissue type [25]. To this end, we monitored the number of facial papillomas developing in cohorts of 24 *Hras* ^*+/+*^ and 27 *Hras* ^*-/-*^ KC mice. Visual inspection revealed an obvious increase in the number of facial lesions (Fig 2A) of papilloma pathology (Fig 2B) in *Hras* ^*-/-*^ KC mice. Quantification revealed that there was an average of 0.7 facial papillomas/mouse in the *Hras* ^*+/+*^ KC cohort, but 2.5 in the *Hras* ^*-/-*^ KC cohort, a significant increase of nearly 4-fold in *Hras* ^*-/-*^ KC mice (Fig 2C). Furthermore, while only about half the *Hras* ^*+/+*^ KC mice developed a papilloma by the study endpoint, all the *Hras* ^*-/-*^ KC mice developed at least one papilloma (Fig 2D). PCR of DNA from a subset of these papillomas revealed the expected recombination of the *LSL-Kras* ^*G12D*^ allele (S1 Fig). Perhaps most telling, the average time when these lesions first appeared in *Hras* ^*+/+*^ KPC mice was 123 days, but 80.5 days in *Hras* ^*-/-*^ KPC mice, a significant decrease of 42.5 days in *Hras* ^*-/-*^ KPC mice (Fig 2D). Similarly, vulvar papillomas also appeared, on average, 8 days earlier in *Hras* ^*-/-*^ KPC mice (Fig 2E). Taken together, these results suggest that loss of Hras promotes oncogenic Kras-driven skin tumorigenesis, potentially at a very early stage such as initiation.

### Loss of wild-type *Hras* affects early pancreatic tumorigenesis

**Fig 3:**
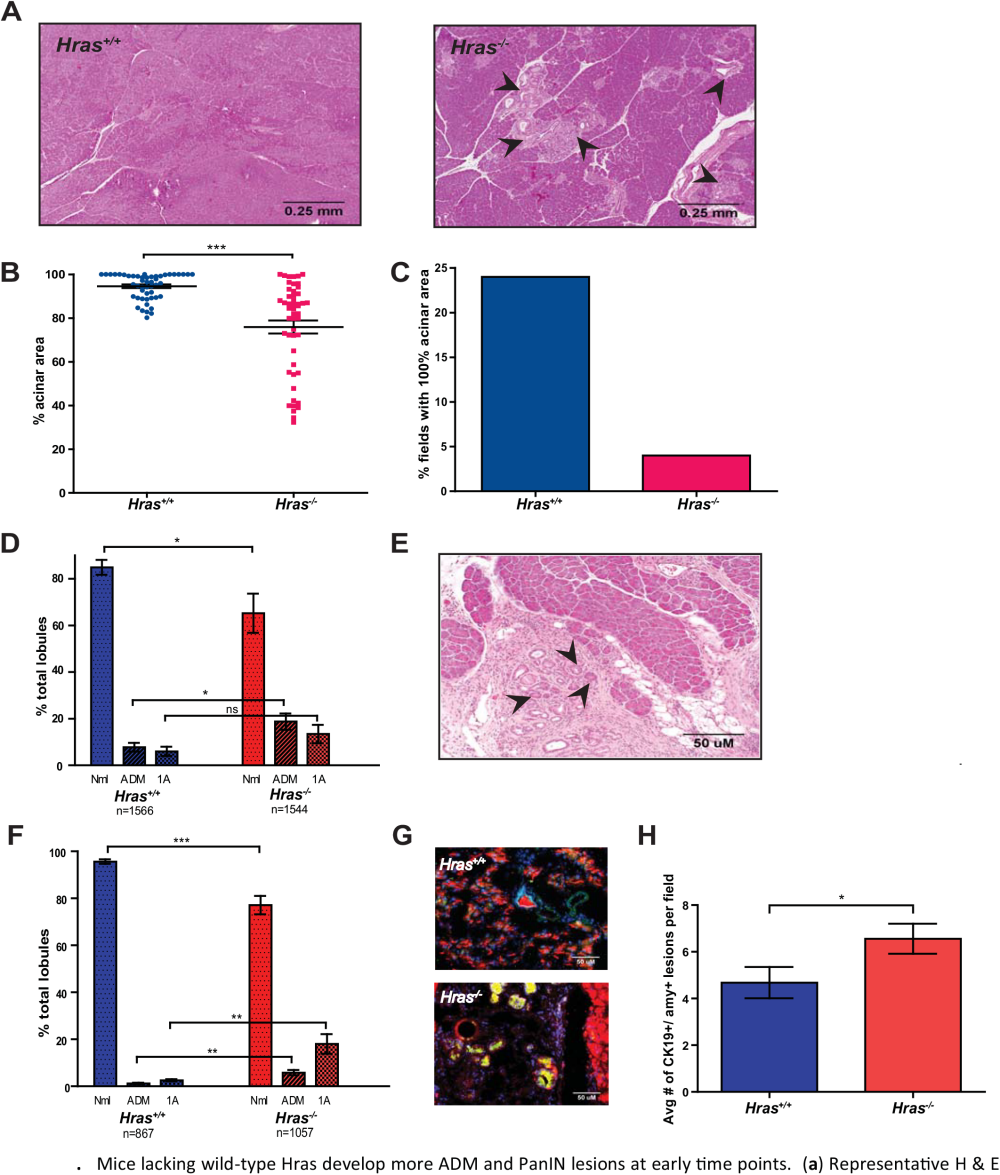
Mice lacking wild-type *Hras* develop more ADM and PanIN lesions at early time points. (**A**) Representative H & E stained section (arrowhead: PanIN lesion), (**B**) quantification of % total normal acinar area remaining per field (at 4x magnification, 5 fields from 10 mice, bar: mean ± S.E.M.), (**C**) % of fields with all normal acinar tissue (from **B**), and (**D**) % of lobules with the indicated highest grade lesion (Nml: normal, ADM: acinar-to-ductal metaplasia, 1A: PanIN-1A, bar: mean ± S.E.M.) of pancreata isolated from *Hras* ^*+/+*^ versus *Hras* ^*-/-*^ KC mice at 8 weeks of age. (**E**) Representative H & E stained section of a pancreas from an 8-week old KC mouse (arrowhead: acinar-to-ductal metaplasia). (**F**) % of lobules with the indicated highest grade lesion (Nml: normal, ADM: acinar-to-ductal metaplasia, 1A: PanIN-1A bar: mean ± S.E.M.) of pancreata isolated from *Hras* ^*+/+*^ versus *Hras* ^*-/-*^ KC mice at 4 weeks of age. (**G**) Representative pancreatic section from a 4-week old *Hras* ^*+/+*^ versus *Hras* ^*-/-*^ KC mouse immunostained for cells (DAPI, blue) and markers of acinar (amylase, red) and ductal (CK-19, green) cells to highlight ADM lesions (co-staining, yellow). (**H**) Number of amylase^+^/CK-19^+^ positive (ADM) cells per field (at 4x magnification, (at 4x magnification, 5 fields from 10 mice) from pancreata isolated from *Hras* ^*+/+*^ versus *Hras* ^*-/-*^ KC mice at 4 weeks of age (bar: mean ± S.E.M.). *P<0.05. ***P<0.0001.

The above observations point towards the *Hras* null background affecting an early stage of tumorigenesis, such as initiation. To assess whether this may explain the observed increase in pancreatic lesions in KC mice and reduced lifespan in KPC mice upon loss of wild-type *Hras*, pancreatic tumorigenesis was analyzed at 8 weeks of age, when KC mice typically exhibit fewer and lower grade lesions [5]. H & E staining of pancreatic sections again revealed a reduction in normal tissue and expansion of PanIN lesions in the *Hras* ^*-/-*^ KC mice (Fig 3A). Quantification of the amount of normal acinar tissue revealed that *Hras* ^*+/+*^ KC mice had an average of 94.6% normal tissue/field, whereas *Hras* ^*-/-*^ KC mice had 75.9%, a significant reduction by nearly 20% in *Hras* ^*-/-*^ KC mice (Fig 3B). This effect was particularly evident when the analysis was repeated by quantitating the number of sections with all the normal acinar tissue remaining. 24% of the sections in *Hras* ^*+/+*^ KC mice had all normal acinar tissue, compared to 4% in *Hras* ^*-/-*^ KC mice, a reduction of 6-fold in *Hras* ^*-/-*^ KC mice (Fig 3C). Pathological grading of 1566 pancreatic lobes from *Hras* ^*+/+*^ KC mice and 1544 pancreatic lobes from *Hras* ^*-/-*^ KC mice revealed 18.7% more lobes with no lesions and a trend towards more PanIN 1A lesions in *Hras* ^*-/-*^ KC mice (Fig 3D). Interestingly, pathological grading also revealed a significant increase of over 2-fold in the number of lobules with acinar-to-ductal metaplasia (ADM) as the highest-grade lesion in *Hras* ^*-/-*^ KC mice (Fig 3D). ADM (*e*.*g*. Fig 3E) is one of the earliest changes in the pancreas of PDAC mouse models, and has been suggested to be a possible precursor to PanIN lesions [28, 29].

Given the increase in the early grade lesions at 8 weeks in *Hras* ^*-/-*^ KC mice, we explored the impact of the *Hras* null genotype at 4 weeks of age when typically there is little or no evidence of pancreatic lesions in KC mice. Pathological grading of 867 lobes from *Hras* ^*+/+*^ KC mice revealed, as expected, nearly all the lobes (95.6%) lacked any lesion (Fig 3F). In contrast, there was a significant increase of nearly 5-fold in the number of lobes with highest grade of ADM lesions and nearly 7-fold in the number of lobes with PanIN 1A lesions in the *Hras* ^*-/-*^ KC mice (Fig 3F). This difference was not recapitulated in culture, as the number of ADM events was similar between cultured *Hras* ^*-/-*^ versus *Hras* ^*+/+*^ acinar cells when *LSL-Kras* ^*G12D*^ was activated by Ad-Cre (not shown). However, we independently and directly validated the increase in ADM lesions *in vivo* in the *Hras* ^*-/-*^ KC mice by quantitating the number of cells co-staining for immunoflourescent markers of acinar (amylase) and ductal (CK-19) cells (Fig 3G). Quantification revealed an average of 4.7 amylase/CK-19 co-staining pancreatic lesions/field in *Hras* ^*+/+*^ KC mice, but 6.6 in *Hras* ^*-/-*^ KC mice, a significant increase in *Hras* ^*-/-*^ KC mice (Fig 3H). There were no differences in the amount of Ki67 (a marker of cellular proliferation), CC3 (a marker of apoptosis), SA-β-gal or p16 (markers of senescence) positive-staining lesions between *Hras* ^*+/+*^ and *Hras* ^*-/-*^ KC mice (S2A–S2D Fig), suggesting that the difference imparted by the loss of Hras had already occurred by the time lesions were detected. Taken together, these data suggest that Hras suppresses early pancreatic tumorigenesis, perhaps at the stage of initiation.

### Wild-type *Hras* is activated in PDAC

**Fig 4:**
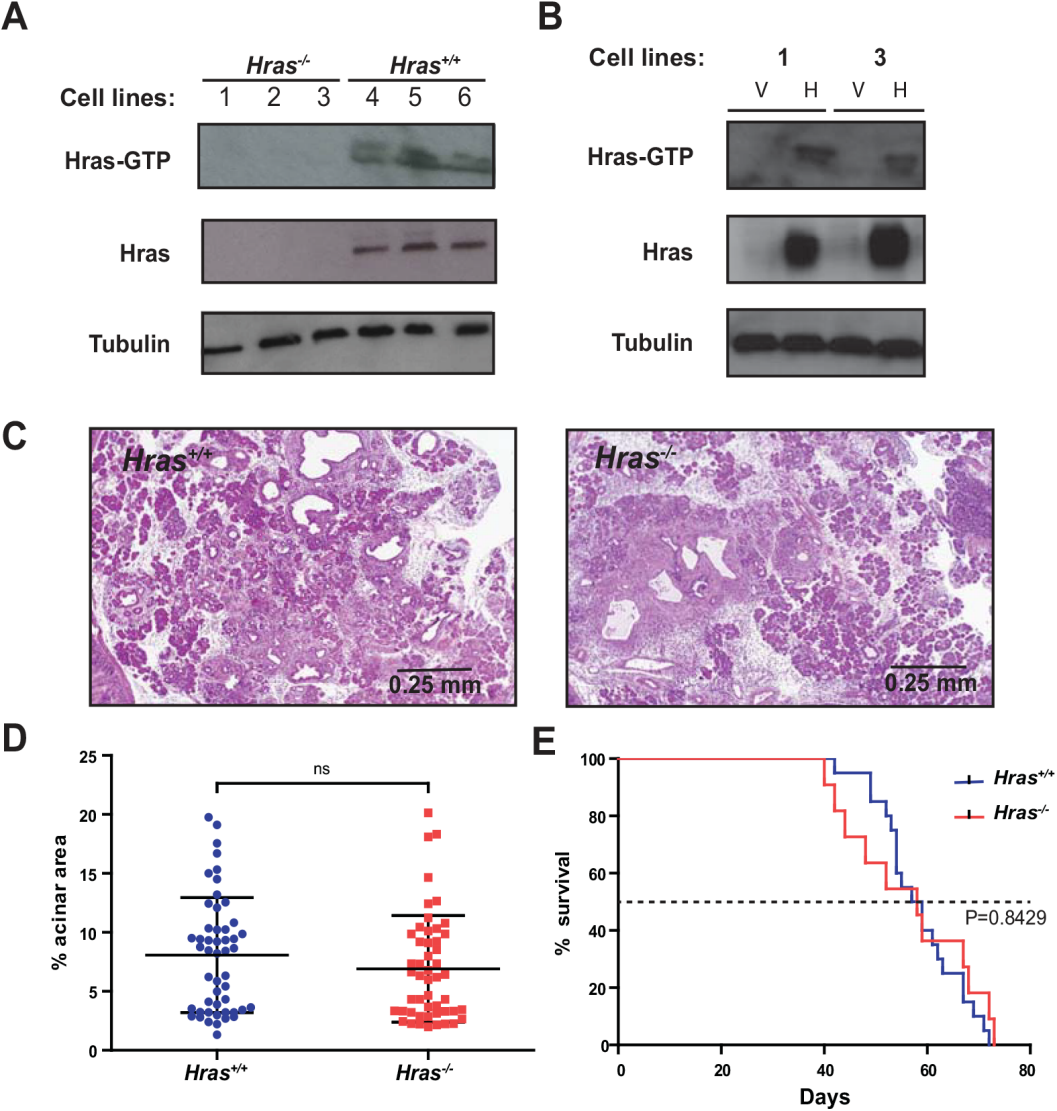
Wild-type Hras is activated in KPC cell lines and *Hras* ^*+/+*^ and *Hras* ^*-/-*^ KPPC mice exhibit similar tumor burden and lifespan. Immunoblot analysis of Hras and Hras-GTP in PDAC cell lines derived from (**A**) *Hras* ^*-/-*^ (cell lines 1–3) and *Hras* ^*+/+*^ (cell lines 4–6) KPC mice or (**B**) PDAC cell lines 1 and 3 derived from *Hras* ^*-/-*^ KPC mice stably infected with a retrovirus encoding no transgene (vector, V) or wild-type Hras (H). Tubulin serves as a loading control. (**C**) Representative H&E stained sections from pancreata of *Hras* ^*+/+*^ versus *Hras* ^*-/-*^ KPPC mice at 14 days of age. (4x magnification) (**D**) Quantification of the % normal acinar area remaining per field (at 4x magnification, 5 fields from 10 mice) from *Hras* ^*+/+*^ versus *Hras* ^*-/-*^ KPPC mice (bar: mean ± S.E.M). (**E**) Kaplan-Meier survival curve of *Hras* ^*+/+*^ (n = 20) versus *Hras* ^*-/-*^ (n = 11) KPPC mice. ns: not significant.

Wild-type Ras can be activated in the presence of a mutant oncogenic allele in cancer cell lines [8–10, 19]. In pancreatic tissue at 8 weeks of age, when there are few PanIN lesions in *Hras* ^*+/+*^ KC mice (Fig 3A and 3B), Hras was not detected at appreciable levels (not shown). To evaluate the status of Hras in later stages of disease, PDAC cell lines were established from three different *Hras* ^*+/+*^ and *Hras* ^*-/-*^ KPC mice. GTP-bound Hras was then affinity captured with Raf-RBD followed by immunoblotting with an anti-Hras antibody to detect the protein. In all three *Hras* ^*+/+*^ KPC cell lines, Hras-GTP was recovered. In contrast, this pool of active Hras was completely absent in the PDAC tumor cells derived from the three *Hras* ^*-/-*^ KPC mice (Fig 4A). Moreover, this pool of active Ras was restored upon re-expressing wild-type Hras in two *Hras* ^*-/-*^ KPC cell lines at early passage (Fig 4B), although this effect was lost as the cells were passaged (not shown). Thus, loss of Hras removes a pool of active Ras from pancreatic tumor cells.

### Loss of wild-type *Hras* has no overt effect on pancreatic tumorigenesis in a homozygous mutant p53 background

The effect on pancreatic tumorigenesis upon losing Hras in a homozygous *Trp53* ^*R172H*^ background, which has been reported to suppress a loss of pancreatic cells when the *LSL-Kras* ^*G12D*^ allele is activated [30], was next determined. Cohorts of 10 *Hras* ^*+/+*^ versus *Hras* ^*-/-*^ mice in a *Kras* ^*LSL-G12D/+*^ *;Trp53* ^*LSL-R172H/LSL-R172H*^ *;Pdx-1-Cre* ^*tg/+*^ (KPPC) background were euthanized at a fixed time point of 14 days of age, and the amount of normal acinar tissue remaining in the pancreas was assessed (Fig 4C). Quantification of H & E stained pancreatic sections revealed no significant difference in the amount of normal tissue remaining between the two cohorts (Fig 4D). Cohorts of 20 *Hras* ^*+/+*^ and 11 *Hras* ^*-/-*^ KPPC mice were also allowed to age until moribundity endpoints, revealing a nearly identical median survival of 50 and 47.5 days, respectively (Fig 4E). With the caveat that the KPPC background may be so aggressive as to mask any effect on early tumorigenesis caused by the loss of *Hras*, these data are consistent with a model whereby a homozygous mutant p53 background can negate the tumor-suppressive effects of wild-type *Hras* on both pancreatic tumorigenesis and survival.

## Discussion

We report that a homozygous null *Hras* background led to more and higher grade pre-invasive PanIN lesions in the KC mouse model of early pancreatic cancer. In agreement, reducing the levels of wild-type Ras signaling also promotes tumorigenesis in other models of pre-invasive cancer [13, 15, 17, 18]. Multiple lines of evidence point towards these phenotypes being a manifestation of the loss of Hras at a very early stage of pancreatic tumorigenesis. First, the percentages of proliferation, apoptosis, and senescence marker-positive PanIN lesions was similar between *Hras* ^*-/-*^ and *Hras* ^*+/+*^ KC mice at an early time point, suggestive of a difference prior to the detection of these lesions. Second, skin papillomas were detected earlier and at a higher frequency in the *Hras* ^*-/-*^ background, consistent with an effect on tumor initiation. In agreement, there were more ADM and PanIN1A lesions in *Hras* ^*-/-*^ KC mice at early time points, both of which have been argued to reflect an initiating event [31]. We attempted to assess if this effect occurred at an even earlier time point by assaying for recombination of LSL-EGFP as a surrogate marker for cells with an activated *KrasG12D* allele, but mosaic staining for GFP precluded any meaningful analysis (not shown). Collectively, these results suggest that the loss of Hras enhances the earliest stages of pancreatic tumorigenesis, perhaps even initiation. Taking this concept one step further, perhaps epigenetic differences in levels of Ras proteins may influence whether an oncogenic mutation in *KRAS* leads to pancreatic tumorigenesis. As KPC mice had a reduced lifespan in the *Hras* ^*-/-*^ background, such early effects of wild-type Ras proteins may have lasting repercussions.

While it remains to be determined how wild-type Hras suppresses early pancreatic tumorigenesis, a number of mechanisms are possible. One possibility is that the loss of Hras may reduce the pool of active Ras to a level below that which triggers a growth arrest in response to activation of oncogenic *Kras*, increasing the chances that an oncogenic mutation in *Kras* will initiate tumorigenesis. More specifically, normal cells can be sensitive to high levels of Ras signaling, resulting in senescence rather than proliferation, which can inhibit tumor formation [32, 33]. In this regard, pancreatic cells expressing oncogenic *Kras* have been reported to be selectively lost at two to six weeks of age, apparently a consequence of high Kras signaling [30]. As such, changes that reduce this signaling may promote tumor initiation. In this regard, we demonstrated that loss of Hras reduced the pool of active Ras in pancreatic cancer cells. Bi-allelic loss of wild-type p53 has been shown to rescue the early loss of pancreatic cells upon activation of oncogenic Kras [30]. In agreement, both tumor burden and lifespan were similar between the *Hras* ^*+/+*^ and *Hras* ^*-/-*^ genotypes in a homozygous mutant p53 background of pancreatic cancer. Admittedly, there was no difference in β-galactosidase staining, a common marker for senescence, between *Hras* ^*+/+*^ and *Hras* ^*-/-*^ *KC* mice at 8-weeks of age, and endogenous Hras was not detected by immunoblot in the pancreatic tissue at this time point. A number of other mechanisms are most certainly possible. For example, loss of Hras may reduce apoptosis or increase proliferation. However, as with the case of senescence markers, there was no difference in Ki67, cleaved caspase 3, or TUNEL staining between *Hras* ^*+/+*^ and *Hras* ^*-/-*^ *KC* mice at 8-weeks of age, arguing either against this model or pointing towards an effect at an earlier time point. Alternatively, rather than the loss of Hras reducing the amplitude of Ras signaling, it is also possible that this loss changes the spectrum of this signaling. More specifically, Ras isoforms exhibit differences in their subcellular localizations, which may result in engagement of effector proteins in different locations within the cells [34]. Loss of Hras may thus reduce the diversity of this signaling. It has also been suggested that wild-type Ras proteins may sequester effectors from the oncogenic protein, thereby reducing the amplitude or altering the signaling output, which may manifest as increased tumorigenesis when wild-type Hras is deleted [35]. The loss of Hras during development may lead to an increase in the type of cells that ultimately give rise to pancreatic cancer, due to increased proliferation, decreased apoptosis or senescence, or altered differentiation. Finally, it is even possible that the loss of Hras in the stroma underlies the increase in pancreatic lesions observed in *Hras* ^*-/-*^ *KC* mice.

We fully recognize that the tumor-suppressive effect of wild-type Hras may very well be context dependent. Indeed, loss of *Nras* has been shown to promote oncogenic Kras-driven lung tumorigenesis, yet inhibit oncogenic Hras-driven skin tumorigenesis [17]. Similarly, two oncogenic alleles of *Nras* were found to be more tumorigenic than one allele in a mouse model of CMML and JMML [36]. The negative impact of wild-type Ras proteins on tumorigenesis may also be negated during tumor progression, for example, when cells become resistant to higher levels of Ras signaling. Wild-type Ras proteins may even promote more malignant stages of tumorigenesis, as shRNA-mediated knock down of wild-type Ras proteins has been shown to inhibit the tumor growth of *KRAS* mutation-positive cancer cell lines isolated from late stage disease [8–10, 19]. Nevertheless, we find that Hras is clearly tumor suppressive in pancreatic cancer, at least at early stages of this disease, which could have important ramifications on the susceptibility of developing pancreatic cancer upon oncogenic insult.

## Supporting Information

S1 Fig The *LSL-Kras* ^*G12D*^ allele is recombined in the pancreas and skin papillomas of KC mice. Representative PCR amplification using primers specific for the wild-type *Kras* allele or the LSL-*Kras* ^*G12D*^ allele following Cre-excision (loxed allele) shows successful recombination in the pancreata (panc) and facial papillomas (pap), but not the negative control tails. DNA was isolated from the tissues of 9-month old *Hras* ^*+/+*^ and *Hras* ^*-/-*^ mice.
(PDF)

S2 Fig Immunohistochemical analysis of lesions from 8-week old KC mice. Representative stained sections and quantification of % positive-staining lesions for (**A**) Ki67 as a marker for proliferation, (**B**) CC3 as a marker for apoptosis, (**C**) p16 and, (**D**) SA-β-gal as markers for senescence. For each, the % of positive-staining lesions was quantified in 10 random high-power fields from 5 mice of each cohort. (bar: mean ± S.E.M.). ns = not significant. Line = 50 μM.
(PDF)

## References

[1] Surveillance, Epidemiology, and End Results [SEER] Program. National Cancer Institute, 2013 Available: www.seercancer.gov.

[2] Jones S, Zhang X, Parsons DW, Lin JC, Leary RJ, Angenendt P, et al Core signaling pathways in human pancreatic cancers revealed by global genomic analyses. Science. 2008; 321: 1801–1806. 10.1126/science.1164368 18772397 PMC2848990

[3] Pylayeva-Gupta Y, Grabocka E, Bar-Sagi D. RAS oncogenes: weaving a tumorigenic web. Nat Rev Cancer. 2011; 11: 761–774. 10.1038/nrc3106 21993244 PMC3632399

[4] Eser S, Schnieke A, Schneider G, Saur D. Oncogenic KRAS signalling in pancreatic cancer. Br J Cancer. 2014; 111: 817–822. 10.1038/bjc.2014.215 24755884 PMC4150259

[5] Hingorani SR, Petricoin EF, Maitra A, Rajapakse V, King C, Jacobetz MA, et al Preinvasive and invasive ductal pancreatic cancer and its early detection in the mouse. Cancer Cell. 2003; 4: 437–450. 14706336 10.1016/s1535-6108(03)00309-x

[6] Huang DC, Marshall CJ, Hancock JF. Plasma membrane-targeted ras GTPase-activating protein is a potent suppressor of p21ras function. Mol Cell Biol. 1993; 13: 2420–2431. 8455619 10.1128/mcb.13.4.2420-2431.1993 PMC359563

[7] Hamilton M, Wolfman A. Ha-ras and N-ras regulate MAPK activity by distinct mechanisms in vivo. Oncogene. 1998; 16: 1417–1428. 9525741 10.1038/sj.onc.1201653

[8] Lim KH, Ancrile BB, Kashatus DF, Counter CM. Tumour maintenance is mediated by eNOS. Nature. 2008; 452: 646–649. 10.1038/nature06778 18344980 PMC2688829

[9] Jeng HH, Taylor LJ, Bar-Sagi D. Sos-mediated cross-activation of wild-type Ras by oncogenic Ras is essential for tumorigenesis. Nat Comun. 2012; 3: 1168. 10.1038/ncomms2173 PMC3640996 23132018

[10] Young A, Lou D, McCormick F. Oncogenic and wild-type Ras play divergent roles in the regulation of mitogen-activated protein kinase signaling. Cancer Discov. 2013; 3: 112–123. 10.1158/2159-8290.CD-12-0231 23103856

[11] Collado M, Serrano M. Senescence in tumours: evidence from mice and humans. Nat Rev Cancer. 2010; 10: 51–517. 10.1038/nrc2772 20029423 PMC3672965

[12] Sarkisian CJ, Keister BA, Stairs DB, Boxer RB, Moody SE, Chodosh LA. Dose-dependent oncogene-induced senescence in vivo and its evasion during mammary tumorigenesis. Nat Cell Biol. 2007; 9: 493–505. 17450133 10.1038/ncb1567

[13] Balmain A, Brown K, Bremner R. The interplay between ras oncogenes and tumor suppressor genes in experimental carcinogenesis. Immunology Ser. 1990; 51: 75–788. 2128916

[14] Hegi ME, Devereux TR, Dietrich WF, Cochran CJ, Lander ES, Foley JF, et al Allelotype analysis of mouse lung carcinomas reveals frequent allelic losses on chromosome 4 and an association between allelic imbalances on chromosome 6 and K-ras activation. Cancer Res. 1994; 54: 6257–6264. 7954475

[15] Zhang Z, Wang Y, Vikis HG, Johnson L, Liu G, Li J, et al Wildtype Kras2 can inhibit lung carcinogenesis in mice. Nat Genet. 2001; 29: 25–33. 11528387 10.1038/ng721

[16] Li J, Zhang Z, Dai Z, Plass C, Morrison C, Wang Y, et al LOH of chromosome 12p correlates with Kras2 mutation in non-small cell lung cancer. Oncogene. 2003; 22: 1243–1246. 12606951 10.1038/sj.onc.1206192 PMC3438910

[17] To MD, Rosario RD, Westcott PM, Banta KL, Balmain A. Interactions between wild-type and mutant Ras genes in lung and skin carcinogenesis. Oncogene. 2013; 32: 4028–4033. 10.1038/onc.2012.404 22945650 PMC3515692

[18] Diaz R, Ahn D, Lopez-Barcons L, Malumbres M, Perez de Castro I, Lue J, et al The N-ras proto-oncogene can suppress the malignant phenotype in the presence or absence of its oncogene. Cancer Res. 2002; 62: 4514–4518. 12154063

[19] Grabocka E, Pylayeva-Gupta Y, Jones MJ, Lubkov V, Yemanaberhan E, Taylor L, et al Wild-type H- and N-Ras promote mutant K-Ras-driven tumorigenesis by modulating the DNA damage response. Cancer Cell. 2014; 25: 243–256. 10.1016/j.ccr.2014.01.005 24525237 PMC4063560

[20] Debacq-Chainiaux F, Erusalimsky JD, Campisi J, Toussaint O. Protocols to detect senescence-associated β-galactosidase [SA-βgal] activity, a biomarker of senescent cells in culture and in vivo. Nat Protoc. 2009; 4: 1798–1806. 10.1038/nprot.2009.191 20010931

[21] de Rooij J, Bos JL. Minimal Ras-binding domain of Raf1 can be used as an activation-specific probe for Ras. Oncogene. 1997; 14: 623–625. 9053862 10.1038/sj.onc.1201005

[22] Esteban LM, Vicario-Abejon C, Fernandez-Salguero P, Fernandez-Medarde A, Swaminathan N, Yienger K, et al Targeted genomic disruption of H-ras and N-ras, individually or in combination, reveals the dispensability of both loci for mouse growth and development. Mol Cell Biol. 2001; 21: 1444–1452. 11238881 10.1128/MCB.21.5.1444-1452.2001 PMC86690

[23] Vincent A, Herman J, Schulick R, Hruban RH, Goggins M. Pancreatic cancer. Lancet. 2011; 378: 607–620. 10.1016/S0140-6736(10)62307-0 21620466 PMC3062508

[24] Hingorani SR, Wang L, Multani AS, Combs C, Deramaudt TB, Hruban RH, et al Trp53^R172H^ and Kras^G12D^ cooperate to promote chromosomal instability and widely metastatic pancreatic ductal adenocarcinoma in mice. Cancer Cell. 2005; 7: 469–483. 15894267 10.1016/j.ccr.2005.04.023

[25] Lampson BL, Kendall SD, Ancrile BB, Morrison MM, Shealy MJ, Barrientos KS, et al Targeting eNOS in pancreatic cancer. Cancer Res. 2012; 72: 4472–4482. 10.1158/0008-5472.CAN-12-0057 22738914 PMC3749841

[26] Mazur PK, Gruner BM, Nakhai H, Sipos B, Zimber-Strobl U, Strobl LJ, et al Identification of epidermal Pdx1 expression discloses different roles of Notch1 and Notch2 in murine Kras[G12D]-induced skin carcinogenesis in vivo. PLoS One. 2010; 5: e13578 10.1371/journal.pone.0013578 21042537 PMC2962652

[27] Gades NM, Ohash A, Mills LD, Rowley MA, Predmore KS, Marler RJ, et al Spontaneous vulvar papillomas in a colony of mice used for pancreatic cancer research. Comp Med. 2008; 58: 271–275. 18589869 PMC2704118

[28] Shi G, DiRenzo D, Qu C, Barney D, Miley D, Konieczny SF. Maintenance of acinar cell organization is critical to preventing Kras-induced acinar-ductal metaplasia. Oncogene. 2013; 32: 1950–1958. 10.1038/onc.2012.210 22665051 PMC3435479

[29] Hill R, Calvopina JH, Kim C, Wang Y, Dawson DW, Donahue TR, et al PTEN loss accelerates Kras^G12D^-induced pancreatic cancer development. Cancer Res. 2010; 70: 7114–7124. 10.1158/0008-5472.CAN-10-1649 20807812 PMC2940963

[30] Morton JP, Timpson P, Karim SA, Ridgway RA, Athineos D, Doyle B, et al Mutant p53 drives metastasis and overcomes growth arrest/senescence in pancreatic cancer. Proc Natl Acad Sci USA. 2010; 107: 246–251. 10.1073/pnas.0908428107 20018721 PMC2806749

[31] Reichert M, Rustgi AK. Pancreatic ductal cells in development, regeneration, and neoplasia. J Clin Invest. 2011; 121: 4572–4578. 10.1172/JCI57131 22133881 PMC3225990

[32] Serrano M, Lin AW, McCurrach ME, Beach D, Lowe SW. Oncogenic ras provokes premature cell senescence associated with accumulation of p53 and p16INK4a. Cell. 1997; 88: 593–602. 9054499 10.1016/s0092-8674(00)81902-9

[33] Collado M, Gil J, Efeyan A, Guerra C, Schuhmacher AJ, Barradas M, et al Tumour biology: Senescence in premalignant tumours. Nature. 2005; 436: 642 16079833 10.1038/436642a

[34] Henis YI, Hancock JF, Prior IA. Ras acylation, compartmentalization, and signaling nanoclusters (Review). Mol Membr Biol. 2009; 26: 80–92. 10.1080/09687680802649582 19115142 PMC2782584

[35] Singh A, Sowjanya AP, Ramakrishna G. The wild type Ras road ahead. FASEB J. 2005; 19: 161–169. 15677339 10.1096/fj.04-2584hyp

[36] Xu J, Haigis KM, Firestone AJ, McNerney ME, Li Q, Davis E, et al Dominant role of oncogene dosage and absence of tumor suppressor activity in Nras-driven hematopoietic transformation. Cancer Discov. 2013; 3: 993–1001. 10.1158/2159-8290.CD-13-0096 23733505 PMC3770749

