# Supplementary figures and images for: Wild-Type Hras Suppresses the Earliest Stages of Tumorigenesis in a Genetically Engineered Mouse Model of Pancreatic Cancer
Source: PLoS One. 2015 Oct 9;10(10):e0140253. doi: 10.1371/journal.pone.0140253 (PMC4599940; doi:10.1371/journal.pone.0140253)

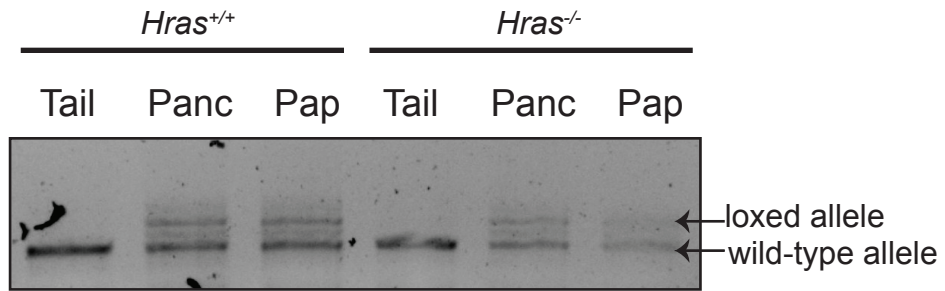

Supplement: S1 Fig — Representative PCR amplification using primers specific for the wild-type Kras allele or the LSL-Kras G12D allele following Cre-excision (loxed allele) shows successful recombination in the pancreata (panc) and facial papillomas (pap), but not the negative control tails. DNA was isolated from the tissues of 9-month old Hras +/+ and Hras -/- mice. (PDF) [file pone.0140253.s001.pdf]

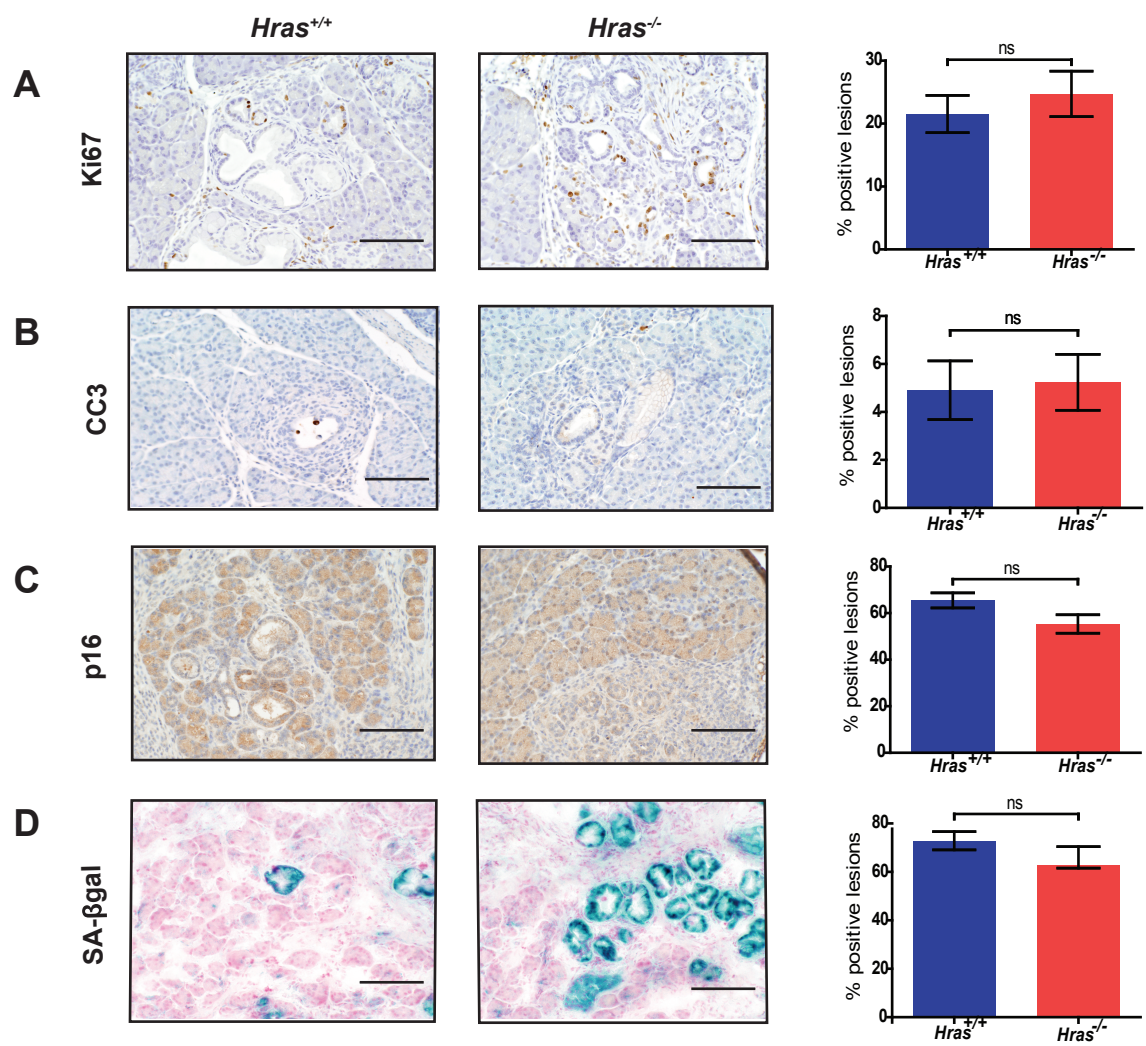

Supplement: S2 Fig — Representative stained sections and quantification of % positive-staining lesions for (A) Ki67 as a marker for proliferation, (B) CC3 as a marker for apoptosis, (C) p16 and, (D) SA-β-gal as markers for senescence. For each, the % of positive-staining lesions was quantified in 10 random high-power fields from 5 mice of each cohort. (bar: mean ± S.E.M.). ns = not significant. Line = 50 μM. (PDF) [file pone.0140253.s002.pdf]
